# A Pilot Study on the Feasibility of Robot-Aided Leg Motor Training to Facilitate Active Participation

**DOI:** 10.1371/journal.pone.0077370

**Published:** 2013-10-11

**Authors:** Chandramouli Krishnan, Rajiv Ranganathan, Yasin Y. Dhaher, William Z. Rymer

**Affiliations:** 1 Department of Physical Medicine and Rehabilitation, University of Michigan Medical School, Ann Arbor, Michigan, United States of America; 2 Department of Physical Medicine and Rehabilitation, Northwestern University Feinberg School of Medicine, Chicago, Illinois, United States of America; 3 Sensory Motor Performance Program, Rehabilitation Institute of Chicago, Illinois, United States of America; University of Cambridge, United Kingdom

## Abstract

Robot-aided gait therapy offers a promising approach towards improving gait function in individuals with neurological disorders such as stroke or spinal cord injury. However, incorporation of appropriate control strategies is essential for actively engaging the patient in the therapeutic process. Although several control algorithms (such as assist-as-needed and error augmentation) have been proposed to improve active patient participation, we hypothesize that the therapeutic benefits of these control algorithms can be greatly enhanced if combined with a motor learning task to facilitate neural reorganization and motor recovery. Here, we describe an active robotic training approach (patient-cooperative robotic gait training combined with a motor learning task) using the Lokomat and pilot-tested whether this approach can enhance active patient participation during training. Six neurologically intact adults and three chronic stroke survivors participated in this pilot feasibility study. Participants walked in a Lokomat while simultaneously performing a foot target-tracking task that necessitated greater hip and knee flexion during the swing phase of the gait. We computed the changes in tracking error as a measure of motor performance and changes in muscle activation as a measure of active subject participation. Repeated practice of the motor-learning task resulted in significant reductions in target-tracking error in all subjects. Muscle activation was also significantly higher during active robotic training compared to simply walking in the robot. The data from stroke participants also showed a trend similar to neurologically intact participants. These findings provide a proof-of-concept demonstration that combining robotic gait training with a motor learning task enhances active participation.

## Introduction

Physical therapy is an important component of recovery after a neurological injury, such as stroke or spinal cord injury [1]. With the growing aging population and associated ailments, there is a great demand for physical therapy services. Using robots to assist in providing therapy is a promising approach to meet this heightened demand [2].

There are several advantages in using robots to assist during therapy. First, the dosage of therapy, which is a strong determinant of neural plasticity and recovery, can be substantially increased. Second, therapy can be provided in diverse and novel dynamic environments, which may be critical for generalizing motor recovery to tasks other than those that are practiced. Third, the physical burden on the therapist can be greatly alleviated thereby minimizing work-related injuries. Finally, patient performance and progression can be objectively monitored using sensors built into the robot.

While there are advantages in using a robot, there are also challenges. A major challenge for robots is the need to at least partly emulate the skills of a trained therapist [3]. Therapists, for example, can assess each patient’s physical capacity and scale their assistance based on the need. They can also provide resistance to movements when appropriate. However, most robots are designed to provide constant assistance without taking into account each patient’s functional ability. As a result, therapy is not tailored to each patient’s need and the ability of a robot to induce neural plasticity is potentially negated.

One approach to overcome this limitation is to actively engage the patient in the robotic training process. Researchers have attempted to achieve this goal by incorporating control algorithms that require the patient to actively initiate movements to perform the task [4]. A good example is the development of patient-cooperative control algorithm for the gait rehabilitation robot called as the Lokomat [5], [6]. This algorithm allows the user to control the amount of assistance provided by the robot during the training process. Therefore, by reducing the amount of guidance provided by the robot, the participant can be encouraged to initiate active movements.

In addition to developing appropriate control algorithms, another important component of therapy is to take advantage of the plasticity in the nervous system. The corticospinal system has the remarkable ability to undergo structural and functional alterations in response to motor training [7], [8], [9], [10]. However, repetitive motor activity alone is not sufficient to drive cortical reorganization. Ideally, motor training should also involve skill acquisition to induce neuronal plasticity that is indicative of motor recovery [11]. Therefore, combining robotic interventions with skill learning tasks is expected to augment cortical plasticity and motor recovery. While the concept of combining robotic training with a motor learning task to improve active participation has been studied in the upper extremity [2], [12], there is a paucity of research on this issue in the lower extremity. Moreover, a large majority of research on the lower extremity have used tasks that are clinically less relevant to improving function (such as single joint movements), thereby questioning its applicability in individuals with neurological disorders [13], [14]. Additionally, the few studies that have combined robotic training with a functionally relevant motor learning task have either used devices that are not commercially available or have utilized suboptimal control algorithms (e.g., gravity compensation) to minimize robot dynamics and interaction forces [15], [16]. Further, it is not clear from these studies whether incorporation of a motor learning task actually improves active participation as motor output or physical effort has not been quantified.

Therefore, the purpose of this study was to test the technical feasibility (i.e., provide evidence of proof-of-concept) of combining Lokomat-assisted walking with a functionally relevant motor learning task in improving short-term motor performance and active participation (note that motor performance refers to the performance in the context of the given motor task and is not to be confused with functional motor recovery). The novelty of the current study is that we used a commercially available gait training robot (i.e., the Lokomat) with sophisticated control algorithms to effectively compensate for robot dynamics while performing the motor learning task. Further, we evaluated muscle activation patterns of several lower-extremity muscles as an indication of active participation. The results of this pilot-study provide evidence that this kind of *active* robotic training facilitates greater participation and is a feasible way of modifying gait patterns when walking in a robotic exoskeleton.

## Materials and Methods

### Participants

Six young adults (5 males and 1 female; Age: 30.4±4.4 years; Height: 1.71±0.04 meters; Weight: 66.4±5.4 kg) with no signs of neurological or orthopedic impairment participated in the study. All participants were right leg dominant as determined by their preferred leg for kicking a ball [17], [18], [19], [20]. In addition, data from 3 male chronic stroke survivors (Age: 51.3±5 years; Height: 1.79±.03 meters; Weight: 86.0±7.4 kg; Stroke duration: 2.4±3.2 years; Lower extremity Fugl-Meyer score: 24.0±7.2) were also collected. The study procedures were approved by the Northwestern University Human Subjects Research Institutional Review Board. Prior to participation, all participants were provided with a brief overview of the study and written informed consent was obtained using a form approved by the Institutional Review Board.

### Experimental Protocol

The experiment was performed on a Lokomat system that incorporated an advanced robotic control algorithm [5], [6]. The Lokomat is a robotic gait training device that has been widely used in the rehabilitation of individuals with neurological disorders. The device is traditionally configured to be operated on a ‘position control mode’ where the robot moves the legs of the participant along a predetermined gait trajectory [21]. In general, the stiffness of the robot is very high in this mode thereby enabling the robot to impose the predefined motions with high repeatability and precision. However, for the same reason, the patient has little influence over the movement trajectories in this control mode.

On the other hand, the advanced control algorithm used here (cooperative control) permits the device to function at low impedance allowing the participant to overcome the forces exerted by the robot. This control algorithm provides the user with the ability to programmatically control the stiffness of the robot (from full guidance to no guidance) using a graphical user interface. When the robot is set to provide 100% guidance, the device will essentially function as a position controlled robot with high impedance (or stiffness), whereas when the robot is set to provide 0% guidance, the device will not provide any assistance to the patient’s movements and will simply compensate for robot dynamics, including inertia.

Prior to the experiment, surface electromyographic (EMG) electrodes (Model MA-311, Motion Labs Systems, Inc., Baton Rouge, LA, USA) were placed over the muscle bellies of vastus medialis (VM), rectus femoris (RF), medial hamstring (MH), lateral hamstring (LH), tibialis anterior (TA), medial gastrocnemius (MG), soleus SO), and gluteus medius (GM) and tightly secured to the skin using self-adhesive tapes and cohesive flexible bandages (CoFlex, Andover Healthcare Inc., Salisbury, MA). The electrodes were placed according to the guidelines established by the international SENIAM initiative (www.seniam.org), except for SO, for which the electrode was placed at 2/3^rd^ of the line between lateral femoral condyle and lateral malleolus located [22]. A common reference electrode was placed over the skin on the dorsum of the hand. The quality of the EMG signals was visually inspected to ensure that the electrodes were appropriately placed and to verify that there were no movement or impact artifacts during walking. The EMG signals were recorded for all the neurologically intact individuals but on only one of the stroke survivors.

The participant’s legs were then attached to the robotic legs using the device’s pelvic strap and thigh and shank cuffs, according to the manufacturer’s guidelines. The participant was positioned in such a way that hip and knee joint axes were closely aligned to the Lokomat’s hip and knee joint axes. The participant then performed maximum voluntary contractions of their hip abductors, knee extensors, knee flexors, ankle dorsiflexors, and ankle plantarflexors against a manually imposed resistance [23]. The maximum contractions were needed for normalizing the EMG data obtained during walking. After a 2-minute rest period, the participant walked on a split-belt ADAL treadmill with embedded force platforms (Techmachine, Andrezieux Boutheon, France) for 5 minutes to orient themselves to the robot and to achieve steady-state behavior.

**Figure 1:**
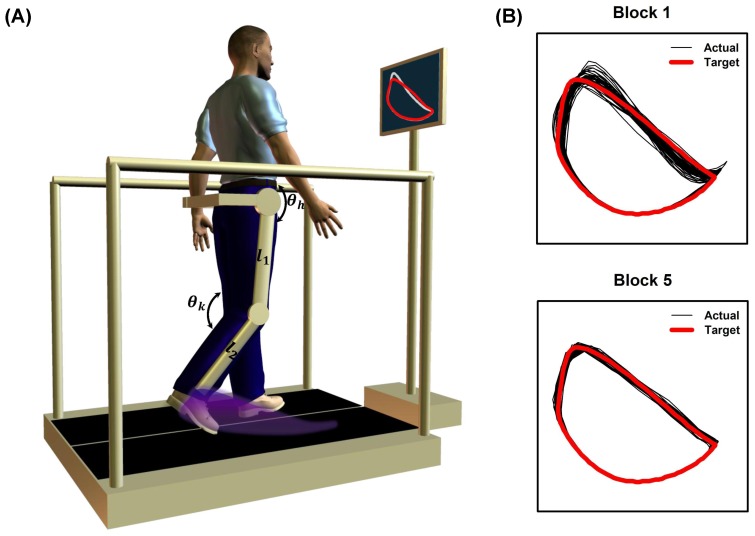
Schematic representation of the active robotic training and a typical example of improvements in target-tracking performance from a neurologically intact participant. (**A**) Schematic representation of the active robotic training paradigm. The training paradigm consisted of two components: (1) walking in the Lokomat that utilized an advanced robotic control algorithm that minimized the amount of guidance provided by the robot and (2) performing a target-tracking task during walking that involved matching their foot trajectory on the sagittal plane (shaded region) to a target-template projected on a monitor placed in front of them. The instantaneous position of the participant’s lateral malleolus, relative to greater trochanter, was computed by performing a forward kinematics on the hip 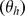 and knee 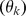 joint angles using the participant’s thigh 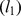 and shank 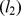 segment length. The target-template corresponded to a gait pattern which required increasing the hip and knee joint angle by a scale of 20% during the swing phase of the gait. (**B**) Example of target-tracking performance from a neurologically intact participant at the beginning (block 1) and end of practice (block 5). Note the increase in target-tracking accuracy with practice.



Following this brief orientation, baseline EMG and kinematic data (hip and knee joint angles) were collected for two minutes when the participant walked in the Lokomat with 10% guidance force. The 10% guidance force was selected based on our pilot work, which suggested that this force adequately compensated the robot dynamics (i.e., inertia) and was most transparent to the subjects without the robot hindering/assisting participant’s intended motion. The kinematic data were recorded using the potentiometers inbuilt in the Lokomat. The baseline kinematic data were ensemble averaged and scaled to generate a target-template trajectory. The target-template trajectory corresponded to a gait pattern which required increasing the hip and knee joint angle by a scale of 20% during the swing phase of the gait. However, the target-template was displayed in the end-point space instead of the joint space, i.e. it was a desired spatial path of the participant’s lateral malleolus of the ankle on the sagittal plane (Figure 1A) [15], [24], [25]. The position of the participant’s ankle lateral malleolus 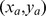, relative to greater trochanter, was obtained by performing a forward kinematics analysis on the hip and knee joint angles using the following equation:
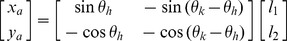
where 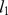 is thigh segment length, 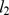 is shank segment length, 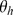 is hip joint angle, and 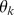 is knee joint angle (Figure 1A). A Hanning window was also used to smooth any abrupt changes in the desired target-template trajectory during the initial and final part of the swing phase. The following formula was used to compute the target-template trajectory from the baseline kinematic data:

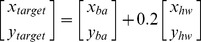
where 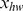, 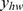 represent the Hanning-windowed version of the baseline trajectories 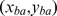. This target-template was then displayed concurrently with the participant’s actual ankle trajectory on a computer monitor placed in front of the participant. The participant was then instructed to match the target continuously for two minutes by modifying the kinematics of their dominant leg (paretic leg in case of the stroke participants). The visual feedback was displayed so that the entire series of ankle positions for one whole gait cycle was visible on the screen (instead of just a single point). The participant performed 5-blocks of target tracking with each block alternated by a 2-minute block of walking with no target-tracking. Thus, the total duration of the training was for about 20 minutes. During the no target-tracking condition, no visual feedback was provided and the participant was instructed to walk in his/her natural pattern.

## Data Analyses

### Target-Tracking Error

In order to evaluate how well participants could track the target template, we computed the target-tracking error for each block. The target-tracking error was computed as the area that was not common to both the reference trajectory (i.e., the target-template) and the actual trajectory during each gait cycle using image processing techniques in MATLAB (Mathworks, Natick, MA). The error values obtained for each gait cycle were averaged to obtain the mean error during target-tracking for a single block. The resulting error was normalized to the error in target-tracking during the 1^st^ block of practice and was expressed as a percentage.

### EMG Processing

To measure active subject participation in our robotic training paradigm, we computed the mean EMG activation of the lower extremity muscles during target-tracking and compared it to the no target-tracking condition. The recorded raw EMG data from baseline and target-tracking trials were band-pass filtered (20–500 Hz), rectified, and smoothed using a zero phase-lag low pass Butterworth digital filter (8^th^ order, 6 Hz Cut-off). The resulting EMG profiles for baseline and target-tracking trials were normalized (using MVC contractions) and averaged across strides and trials to compute mean EMG activity during these conditions. We also divided the gait cycle in to 8 equal phases and computed the average EMG activity during each phase (averaged across strides and trials) to determine the approximate phases during which the changes in EMG activation happened.

### Statistical Analyses

All statistical analyses were performed using SPSS for windows version 20.0 (SPSS Inc., Chicago, IL, USA). A formal statistical analysis was performed only in the control participants and not in the stroke participants. A one-factor repeated measures ANOVA with practice block as a within-subjects factor was performed to detect significant improvements in target-tracking performance with practice. A significant main effect was followed by post-hoc analysis using paired t-tests with Bonferonni correction. EMG data were log transformed (log_e_EMG) prior to statistical analyses to minimize skewness and heteroscedasticity [26], [27]. A two-factor (walking condition and phase) repeated measures ANOVA for each muscle was used to determine significant differences in muscle activation between no target-tracking and target-tracking conditions and to determine whether muscle activation was modulated in a phase dependent manner. A significance level of α = 0.05 was set for statistical analyses. Estimates of effect size were reported using partial η^2^_._

## Results

### Target-tracking Error

**Figure 2:**
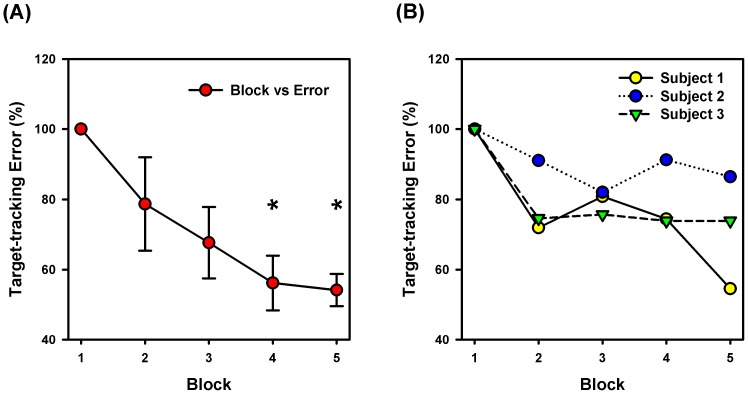
Improvements in target-tracking performance with practice. (**A**) Data from neurologically intact participants demonstrating the reduction in target-tracking error with practice. Target-tracking error is shown as % error observed in block 1. Significant differences were observed between block 1 vs. block 4 and block 1 vs. block 5. (**B**) Changes in target-tracking performance of 3 chronic stroke survivors. Note that two participants showed good reduction in target-tracking error with practice, whereas one participant showed only a modest reduction in tracking-error.

**Figure 3:**
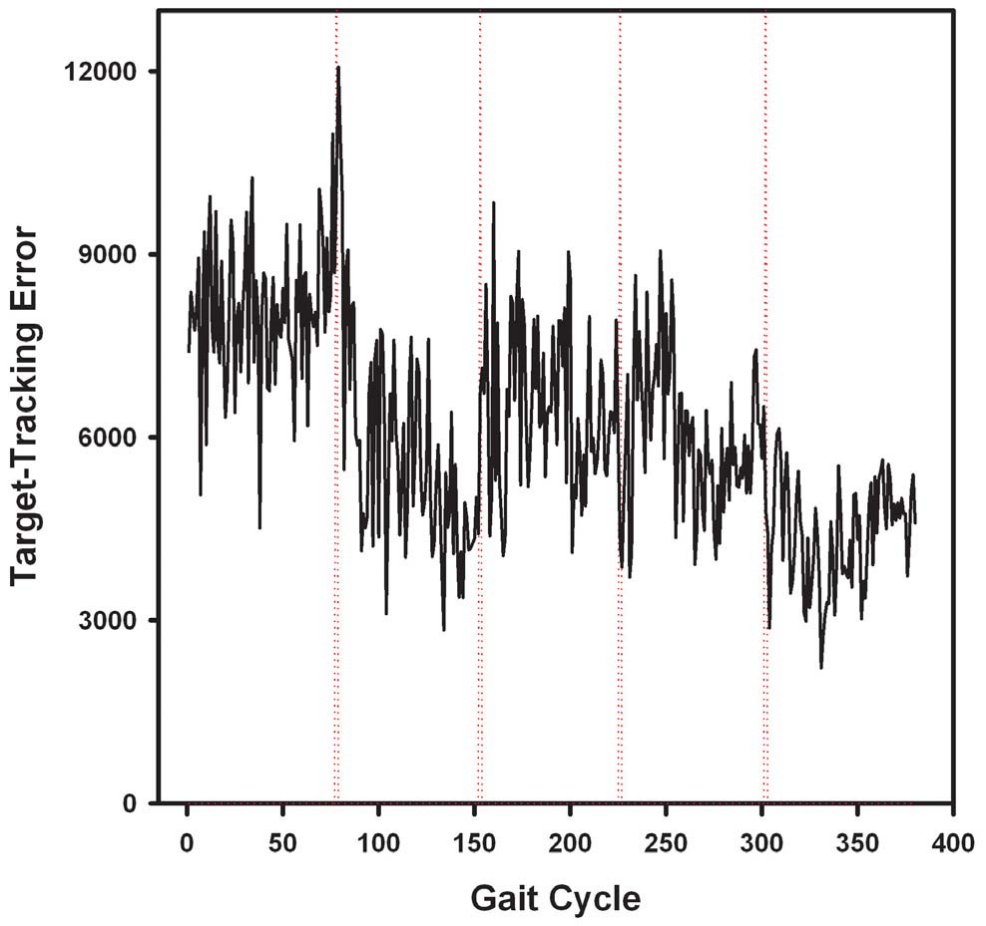
Cycle-by-cycle target-tracking error from a stroke participant. Example of cycle-by-cycle target tracking error from a stroke participant. The dotted vertical lines indicate the end of a practice block.

In the case of control participants, all participants were able to reduce their target-tracking error with practice. An example of the decrease in target-tracking error from a control participant is shown in Figure 1B. Repeated measures ANOVA revealed a significant main effect of practice block on target-tracking error [*F*(4,20) = 9.066; *P*<0.001; partial η^2^ = 0.645; observed power = 0.996] (Figure 2A). Post-hoc analysis indicated that the tracking-error was significantly lower in block 4 (*P* = 0.01) and block 5 (*P* = 0.001) in comparison to block 1 of practice. The mean reduction in tracking error over 5-blocks of practice was 43.6±11.5%. In the case of stroke participants, two of them showed good improvements in target-tracking performance (mean reduction in error 35.8%, Figure 2B), whereas one participant showed only a modest reduction in tracking error with practice (13.5%, Figure 2B). An example of cycle-by-cycle error in target-tracking from a stroke survivor is plotted in Figure 3.

### Muscle Activation during Target-tracking

**Figure 4:**
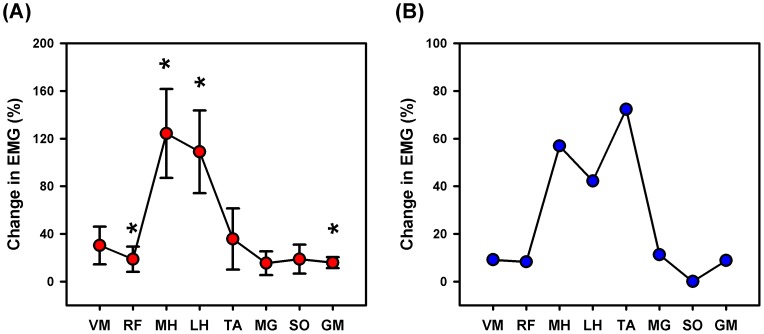
Muscle activation changes during target-tracking. (**A**) Mean muscle activation changes during target-tracking in neurologically intact individuals. In general, higher activation was observed for all the tested muscles. However, statistical significance was obtained only for rectus femoris, medial hamstring, lateral hamstring, and gluteus medius muscles. (**B**) Mean muscle activation changes during target-tracking from a stroke survivor. Note that the muscle activation profiles of the stroke participant during target-tracking showed a trend similar to the control participants, but were less profound, except for the tibialis anterior muscle. VM = vastus medialis, RF = rectus femoris, MH = medial hamstring, LH = lateral hamstring, TA = tibialis anterior, MG = medial gastrocnemius, SO = soleus, and GM = gluteus medius. Changes in muscle activation during target-tracking are shown as a % of no target-tracking condition.

**Figure 5:**
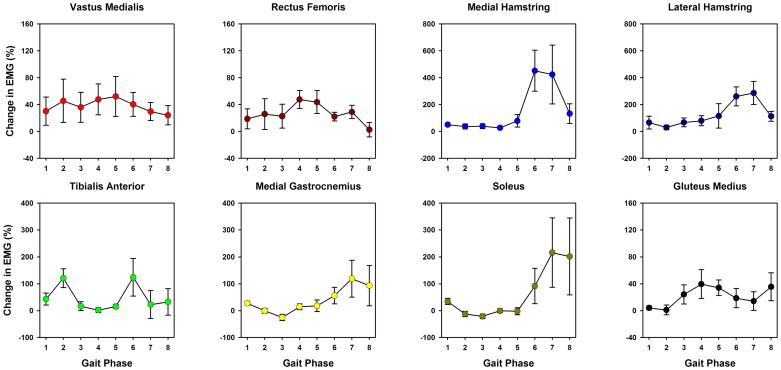
Phase dependent modulation in muscle activity during target-tracking. Mean muscle activation changes during target-tracking examined across 8 equal phases across a stride. Phase dependent modulation in muscle activation was seen for medial hamstring, lateral hamstring, tibialis anterior, and soleus muscles. Although target-tracking required modulation of muscle activity during swing phase of the gait, changes were observed during both stance (Phases 1–5) and swing phases (Phases 6–8) of the gait. Note that the Y-axis scales in the subplots are not equal. Changes in muscle activation during target-tracking are shown as a % of no target-tracking condition.

The mean magnitude of muscle activation was in general higher (21% to 155%) during target-tracking than during no target-tracking condition (Figure 4A). The repeated measures ANOVA indicated that the changes in the magnitude of muscle activity between no target-tracking and target-tracking conditions varied based on the muscle tested. There was a significant main effect (Figure 4A) of walking condition on lower extremity muscle activation for the rectus femoris [*F*(1,5) = 8.021; *P* = 0.037; partial η^2^ = 0.616; observed power = 0.624], medial hamstring [*F*(1,5) = 30.431; *P* = 0.003; partial η^2^ = 0.859; observed power = 0.991], lateral hamstring [*F*(1,5) = 29.786; *P* = 0.003; partial η^2^ = 0.856; observed power = 0.989], and gluteus medius muscles [*F*(1,5) = 14.591; *P* = 0.012; η^2^ = 0.745; observed power = 0.859]. The repeated measures ANOVA also showed a significant condition × phase interaction effect for the medial hamstring [*F*(7,35) = 4.105; *P* = 0.002; partial η^2^ = 0.451; observed power = 0.965], lateral hamstring [*F*(7,35) = 4.332; *P* = 0.002; partial η^2^ = 0.464; observed power = 0.974], tibialis anterior [*F*(7,35) = 2.353; *P* = 0.044; partial η^2^ = 0.320; observed power = 0.773], and soleus muscles [*F*(7,35) = 3.871; *P* = 0.003; partial η^2^ = 0.436; observed power = 0.954], indicating that the modulation in activity of these muscles during target-tracking was phase-dependent (Figure 5). The muscle activation profiles of the stroke participant during target-tracking showed a trend similar to controls, but were less profound, except for the tibialis anterior muscle (Figure 4B).

## Discussion

The purpose of this study was to evaluate the feasibility of incorporating a motor learning task during Lokomat-assisted walking and to test whether short-term motor performance and active patient participation can be enhanced by this training approach. The motor learning task comprised of tracking a target-template that necessitated greater hip and knee motion during the swing phase of the gait. We found that both neurologically intact and stroke participants were able to perform the target-tracking task and improve performance with practice. The target-tracking task also resulted in an increase in active subject participation as evidenced by greater muscle activation during this condition in comparison to the baseline no target-tracking condition.

The inclusion of a motor learning task in the current approach is based on a large body of evidence that suggests that the human brain has a remarkable ability to undergo structural and functional alterations (i.e., neuroplasticity) in response to motor training and skill acquisition [7], [8], [9], [10]. Because neuroplasticity is critical for recovery after a neurological injury, it has been suggested that the same mechanisms underlying motor learning may also contribute to motor recovery after injury [28], [29]. Although the concept of motor learning and its importance to motor recovery has been well studied in the upper extremity [30], [31], [32], [33], [34], this has been relatively overlooked in the lower extremity. This could be possibly due to the fact that walking is considered to be an automatic activity, essentially requiring little cognitive effort [35]. However, there is evidence to support that plastic changes are also possible in the leg motor area following motor skill training [36], [37]. The paradigm described in this study provides an approach to incorporating a skill acquisition task for the lower extremity and opens the opportunity to study the effects of motor learning on neuroplasticity and recovery after stroke. While we recognize that several studies have studied the concept of motor adaptation (a form of motor learning) in a functional task such as gait [38], [39], [40], [41], there have been several lines of evidence that suggest that motor adaptation is qualitatively different from skill acquisition and may contribute less to motor recovery than skill learning [28], [42].

From the perspective of robotic training, active engagement of the patient in the therapeutic process is a key component for optimal recovery after stroke. Studies examining the clinical efficacy of robot-aided gait therapy have shown mixed results [43], [44], [45], [46], [47]. While the results are conflicting, there is a consensus that robot-aided gait training is not demonstrably superior to conventional therapy (when matched for dose) in participants who have preserved ambulatory capacity [48]. Moreover, two recent major clinical trials in sub-acute and chronic stroke survivors revealed that robot-aided gait rehabilitation is substantially inferior to conventional rehabilitation [43], [49]. A pervasive notion in the neuroscience community for the lack of superior outcomes with robot-based interventions is that the control algorithms used to provide therapy do not facilitate active participation and interferes with motor learning [4]. For example, most conventional gait training robots are controlled using position-control algorithms where the robot imposes a predefined gait pattern without taking participant’s volitional effort into account. Studies have shown that excessive guidance from the robot makes the participant tend to remain passive and results in a reduction of physical effort from the participant as the robot provides full guidance to their limb movements [50]. Physical guidance to movements is also known to impair motor learning due to reduced intralimb kinematic variability during locomotor training [51]. The use of patient-cooperative control strategy is known to improve active patient participation (as measured through EMG activation) in the training [52]. Moreover, it also induces larger kinematic variability than a position-control strategy, which may also enhance motor recovery [52]. Here, we show that the addition of a motor learning task on top of a patient-cooperative control strategy further increases active participation by inducing greater muscle activation. Therefore, it is expected that the proposed active robotic training paradigm would augment therapeutic outcomes to a larger extent than the patient-cooperative robotic training, which has already demonstrated some superiority over the conventional robotic training using position control algorithms [52], [53].

It is to be noted that the current study used a target template that was a scaled-up version of the swing phase of the footpath trajectory. We chose this template for three reasons, all of which have clinical implications: First, using a pattern that increases hip and knee flexion will increase active participation and prevent “motor slacking” (i.e., letting the robot do all the work). Second, because we only scale up the swing phase (and not the stance phase), the successful matching of the template requires decoupling of the hip and knee joint motions during the latter part of swing phase (extending the knee while maintaining hip flexion). Training with a pattern that requires this decoupling has potential implications for overcoming the abnormal muscle synergy that is observed after stroke. It is also important to note that the transition between the original stance phase motion and the “modified” swing phase motion is not abrupt because of the smoothing by the Hanning window. Finally, from a clinical perspective, producing greater hip and knee flexion during the swing phase of the gait has direct implications to the characteristic stiff-knee gait observed after stroke. However, it is worth noting that the template can be customized according to the need of the patient’s impairment. For example, a common impairment in stroke patients is limited hip extension during stance phase of the gait. In this case, a target-template that was created by scaling the hip angle during the late stance phase of the gait would be ideal for training.

The present study utilized a target-tracking template that primarily required greater hip and knee flexor muscle activation during the swing phase of walking. Accordingly, we observed substantial EMG increases in the medial and lateral hamstring muscles during initial- and mid-swing phases (gait phases 6 and 7, Figure 5). In addition, we also observed phase-dependent muscle activation changes even in many non-targeted muscles. The tibialis anterior muscle showed higher activation both during the early stance and initial swing phases (gait phases 2 and 6 respectively, Figure 5) and the soleus muscle showed higher activation during the mid- and terminal-swing (gait phases 7 and 8, Figure 5). Although we were not able to record ankle angle in the Lokomat, the changes in muscle activity during target-tracking seem to resemble changes in muscle activity observed during obstacle crossing tasks (such as increased dorsiflexion during initial swing) [54]. The observation of phase-dependent changes in activity of the non-targeted muscles suggests that the target-tracking paradigm requires the coordination between multiple muscles in the lower limb. While the increases in muscle activation in some of the muscles were not substantial, it is expected that even a slight increase in muscle activity over a prolonged period of time will substantially increase the overall muscle work. It is possible that some of the increase in muscle activation simply reflects a global increase in muscle co-contraction observed in early stages of motor learning [55], [56]. However, we believe that this does not account for all the changes for two reasons. First, the relative timing of changes in muscle activation in many muscles was phase-dependent and differed for different muscles and second, we have shown previously in a case report that long-term training using this paradigm resulted in improvements in muscle coordination, resembling patterns observed in control participants [36]. Although the term active participation can also include cognitive factors like motivation and attention, the observed increase in muscle activation of several of the lower extremity muscles provides proof-of-concept that active robotic training paradigm can successfully minimize motor slacking.

There are some limitations to this study. First, the sample size was low, the sample did not include individuals with a recent stroke (i.e., acute/sub-acute), and the participants were younger than the mean age for suffering a stroke. Therefore, we recommend caution when generalizing the study results. Second, we tested only one target-template to verify the feasibility of performing a motor learning task while walking in the Lokomat. As a result, it is not clear whether this template is optimal for improving active participation. It seems that there is an inherent trade-off between increasing active participation and maintaining the duration of training (so as not to induce fatigue) that may need to be further explored. A third limitation is that we did not test the long-term retention of tracking-performance. It would be important to know whether participants can retain their motor performance over longer time periods and whether this ability generalizes to different target-templates, which would suggest an increase in behavioral flexibility [29]. A fourth limitation is that individuals with substantial motor impairments may not be a suitable candidate for this robotic training paradigm as some amount of hip and knee flexor strength is required to perform the target-tracking task. However, these candidates may still benefit by robotic training with reduced guidance as opposed to full guidance [53], which is the current standard of practice in many clinics. Finally, the optimal feedback modality and practice schedule to maximize learning and recovery is still under debate. While we utilized a visual feedback paradigm, others have proposed a force feedback paradigm (such as error augmentation or reduction) to facilitate learning [57]. There is also some evidence to suggest that a combination of visual and force-feedback may have greater effects than either feedback modality alone [16] although there is the possibility that the effects of feedback on learning may be subject-specific.

In summary, this study pilot-tested a robotic training paradigm termed active robotic training to improve short-term locomotor performance and to effectively engage the participant in the training process. Active robotic training comprised of walking in a patient-cooperative robot while simultaneously performing a motor learning task. The results of this pilot study provide proof-of-concept for the feasibility of combining robotic gait training with a motor learning task both in neurologically intact adults and in stroke survivors. We found that the tracking-error, which is indicative of motor performance, was effectively minimized with practice. More importantly, active robotic training also increased EMG activity of several lower extremity muscles indicating that the participants were more actively engaged in the training than during simple patient-cooperative robotic training. Future trials are needed to establish the therapeutic benefits of active robotic training in restoring gait function after stroke.
